# Effects of increased body mass index on one year outcomes following soft tissue arthroscopic shoulder instability repair

**DOI:** 10.1016/j.jseint.2023.05.007

**Published:** 2023-05-27

**Authors:** Aidan G. Papalia, Paul V. Romeo, Neil Gambhir, Matthew G. Alben, Tas Chowdhury, Trevor Simcox, Andrew Rokito, Mandeep S. Virk

**Affiliations:** Division of Shoulder and Elbow Surgery, Department of Orthopedic Surgery, NYU Grossman School of Medicine, NYU Langone Orthopedic Hospital, NYU Langone Health, New York, NY, USA

**Keywords:** Shoulder instability, Arthroscopic stabilization, Bankart repair, Obesity, Shoulder dislocation, Body mass index, Outcomes

## Abstract

**Background:**

The purpose of this study was to investigate the impact of high body mass index on the 1-year minimal outcome following arthroscopic shoulder stabilization.

**Methods:**

Patients who underwent arthroscopic Bankart repair (ABR) between 2017 and 2021 were identified and assigned to 1 of 3 cohorts based on their preoperative body mass index: normal (18-25), overweight (25-30), and obese (>30). The primary outcomes assessed were postoperative shoulder instability and revision rates. The 3 groups were compared using the Patient-Reported Outcomes Measurement Information System (PROMIS) upper extremity, pain interference, pain intensity, Clinical Global Impression scores, visual analog scale pain scores, and shoulder range of motion at 1 year postoperatively.

**Results:**

During the study period, 142 patients underwent ABR and had an average age of 35 ± 10 years. Obese patients had a higher percentage of partial rotator cuff tears (60% vs. 27%, odds ratio: 3.2 [1.1, 9.2]; *P* = .009), longer mean operative time (99.8 ± 40.0 vs. 75.7 ± 28.5 minutes; *P* < .001), and shorter time to complication (0.5 ± 0 vs. 7.0 ± 0 months; *P* = .038). After controlling for confounding factors, obesity was associated with a lesser improvement in upper extremity function scores (obese vs. normal: −4.9 [−9.4, −0.5]; *P* = .029); although this difference exists, found future studies are needed to determine the clinical significance. There were no differences in patient reported outcome measures, recurrence rate, or revision surgery rates between cohorts at any time point (*P* > .05).

**Conclusion:**

Obesity is an independent risk factor for longer operative times but does not confer a higher risk of recurrent instability, revision surgery, or lower outcome scores 1 year following ABR.

Obesity is a growing public health concern in the United States contributing to higher medical expenses and greater comorbidity in affected populations.^9^^,^^40^ With a national obesity prevalence of 42% that is projected to increase to 51% by 2030, it is of the utmost importance to understand the impact that increased body mass index (BMI) has on patient outcomes.^9^^,^^15^ Studies have demonstrated obesity to negatively impact the musculoskeletal system in multiple domains, such as increasing the propensity of osteoarthritis in weight bearing joints, altering the physiological inflammatory response via increased release of pro-inflammatory cytokines, and conferring a higher risk of musculoskeletal injury.^3^^,^^8^^,^^22^^,^^26^ In turn, obesity has been associated with increased rates of perioperative complications following orthopedic surgery.^2^

All-cause shoulder instability occurs in approximately 62.2 per 100,000 people.^2^ Though many patients are successfully treated without surgery, up to 5% require some form of surgical treatment. Surgical options include soft tissue stabilization (Bankart repair and remplissage) or bony procedures (Latarjet, Bristow-Latarjet, and arthroscopic Trillat). Arthroscopic capsulolabral repair (Bankart repair) is the most commonly performed arthroscopic procedure for anterior shoulder instability.^7^^,^^13^^,^^20^ While increased BMI has been implicated as a risk factor for worse outcomes following shoulder surgery, this association has not yet been investigated for arthroscopic soft-tissue stabilization procedures in patients with anterior shoulder instability.^11^^,^^16^^,^^21^ The purpose of this study was to examine the effects of increased BMI on outcomes following arthroscopic Bankart repair (ABR). We hypothesize that patients with increased BMI will report poorer postoperative function, higher pain intensity, and undergo more postoperative dislocations following ABR by 1-year minimal follow-up postoperatively when compared to patients with a normal BMI.

## Methods

### Study design and cohort selection

Following institutional review board approval, we conducted a retrospective analysis of prospectively collected data on patients identified from an institutional database utilizing CPT (Current Procedural Terminology) code 29806 between January 2017 and December 2020. The CPT code was utilized to screen for patients prior to surgery. The inclusion criteria were: (1) age ≥16 years; (2) minimum 1-year follow-up; (3) had completed the preoperative PROMIS (Patient-Reported Outcomes Measurement Information System) upper extremity (P-UE), pain interference (P-Interference), and pain intensity (P-Intensity) surveys; (4) were able to provide informed consent; and (5) elected for primary ABR.^1^^,^^4^^,^^5^^,^^19^^,^^30^^,^^33^^,^^36^^,^^45^ Patients who underwent a revision Bankart procedure or prior ipsilateral shoulder surgery were excluded from analysis. The final follow-up data (PROMIS scores and patient satisfaction) were collected by phone calls, during office visits, or electronically via RedCap (Vanderbilt University, Nashville, TN, USA) where all patient data were securely stored and hosted by our institution. This was done in an effort to limit bias by excluding patients unable or unwilling to complete the outcome measures electronically as studies have shown no differences in outcome scores based on means of completion.^1^^,^^6^

### Perioperative variables measured

BMI was obtained during patients’ preoperative evaluation using standard metrics (height and weight). Subjects were classified into 3 groups based on their baseline BMI: normal (BMI < 25), overweight (BMI ≥ 25 and < 30), and obese (BMI ≥ 30). Demographics (eg, age, sex, and race), concomitant medical conditions, and operative variables (concomitant procedures and procedure time) were also recorded. Glenoid bone loss and Hill-Sachs lesion size were measured on magnetic resonance imaging or computed tomography imaging in order to determine the extent of bone loss and glenoid tracking.^23^^,^^39^^,^^41^^,^^46^, ^47^, ^48^

### Outcomes measured

Shoulder pain and functional outcomes were assessed using the P-Interference, P-Intensity, and P-UE surveys. These surveys were administered both preoperatively and after a minimum of 1-year following surgery. As a validated assessment tool which utilizes computer adaptive testing, PROMIS was employed as a way to reduce survey burden on patients while also allowing an individual's scores to be compared to all test takers.^30^^,^^33^^,^^45^

Patients also reported any perceived change in shoulder pain and functional status after surgery using the Clinical Global Impressions (CGIs) scale, a 7-point scale with options ranging from “much worse” to “much better.”^4^ CGI scale responses were scored from −3 to 3. A score of 1 (“slightly better”) was deemed to be the minimum clinically important difference, while a score of 2 (“better”) was considered to be the substantial clinical benefit (SCB). Free shoulder range of motion (ROM) without scapulothoracic stabilization was measured during the preoperative visit and at the 1-year minimum follow-up visit.

### Statistical methods

Normality of continuous variables was assessed using the Shapiro-Wilk test. Baseline demographics, operative variables, and preoperative PROMIS scores were compared between the BMI groups using the Kruskal-Wallis test and Dwass-Steel-Critchlow-Fligner post hoc testing. A post hoc power analysis was performed (G∗Power Version 3.1.9.7; G∗Power, Heinrich Heine Universität, Düsseldorf, Germany) using a 3.5% difference in postoperative complication rates as the threshold for clinical significance. With the goal of achieving a minimum power of 80% at an alpha equal to 0.05, it was determined that our comparison of postoperative complications between BMI cohorts was underpowered. According to subsequent priori conducted using the same parameters, a minimal sample size of 336 patients per cohort would be necessary in order to detect a 3.5% difference in postoperative complication rates. Therefore, while our study was underpowered with respect to detecting a significant difference in postoperative complication rates, comparisons of complication rates between cohorts are still useful in demonstrating complication trends.

BMI group, age, sex, smoking history (ever-smoker vs. never-smoker), and comorbidities (diabetes, hyperlipidemia, and hypertension) were entered into a multiple linear regression model for continuous outcomes (PROMIS and CGI scores) and a multivariable logistic regression model for binary outcomes (eg, occurrence of any complication). Statistical analysis was performed using Python Jupyter Notebook (Project Jupyter, New York, NY, USA). For all analyses, *P* values < .05 were considered significant.

## Results

A total of 142 patients (111 male, 31 female), average age 35 +/−12 year old, met the inclusion criteria for this study. Patients were segregated into cohorts according to their respective preoperative BMI’s; there were 55 (42 male, 13 female) patients with a normal BMI, 57 patients who were overweight (46 male, 11 female), and 30 patients who were obese (23 male, 7 female). Comparison of patient characteristics and demographics among the 3 groups are shown in Table I. The obese cohort had a higher proportion of patients with diabetes (*P* = .023), hyperlipidemia (*P* = .019), and were older (*P* < .001) compared to patients in other cohorts. The average follow-up for all patients was 27 +/− 8 months (BMI < 25: 27 +/− 9 months, BMI 25-30: 27 +/− 7, and BMI > 30: 27 +/− 8; *P* = .913).

**Table I:** Baseline demographics.

	Overall (n = 142)	BMI < 25 (n = 55)	BMI 25-30 (n = 57)	BMI ≥ 30 (n = 30)	*P* value∗
BMI, mean (SD)	27 ± 5	22 ± 2	27 ± 2	35 ± 4	**<.001**
Age, mean (SD)	35 ± 12	31 ± 12	34 ± 11	42 ± 13	**<.001**
Sex, n (%)					
Male	111 (78%)	42 (76%)	46 (81%)	23 (77%)	.8
Female	31 (22%)	13 (24%)	11 (19%)	7 (23%)	
Race/ethnicity, n (%)					
White	95 (67%)	36 (66%)	40 (70%)	19 (63%)	.6
African American	9 (6%)	2 (4%)	3 (5%)	4 (13%)	
Asian or Pacific Islander	12 (9%)	5 (9%)	5 (9%)	2 (7%)	
Other	26 (18%)	12 (22%)	9 (16%)	5 (17%)	
Procedure, n (%)					
Bankart	85 (60%)	30 (55%)	39 (68%)	16 (53%)	.2
Bankart + capsulorrhaphy	57 (40%)	25 (45%)	18 (32%)	14 (47%)	
Smoking history, n (%)					
Never	103 (73%)	44 (80%)	41 (72%)	18 (60%)	.1
Smoker	39 (28%)	11 (20%)	16 (28%)	12 (40%)	
ASA classification, n (%)					
I	85 (60%)	45 (82%)	31 (55%)	9 (30%)	**<.001**
II	51 (35.9%)	9 (16%)	26 (46%)	16 (53%)	
III	6 (4%)	1 (2%)	0 (0%)	5 (17%)	
Comorbidities, n (%)					
COPD	16 (11%)	8 (15%)	4 (7%)	4 (13%)	.4
Diabetes	4 (3%)	1 (2%)	0 (0%)	3 (10%)	**.023**
Hypertension	14 (10%)	2 (4%)	6 (11%)	6 (20%)	.05
Hyperlipidemia	18 (13%)	3 (6%)	7 (12%)	8 (27%)	**.019**
Cancer	4 (3%)	1 (2%)	0 (0%)	2 (7%)	.3
Preoperative PROMIS scores, mean (SD)					
Pain interference	56 ± 8	56 ± 8	56 ± 8	58 ± 8	.5
Pain intensity	48 ± 8	47 ± 8	47 ± 8	51 ± 5	.07
Upper extremity function	40 ± 9	41 ± 8	39 ± 10	38 ± 8	.09
Preoperative ROM, mean (SD)					
Flexion	160 ± 25	164 ± 28	159 ± 24	157 ± 24	.5
External rotation	62 ± 21	62 ± 21	65 ± 22	56 ± 20	.1
Internal rotation score∗∗	5.9 ± 1.2	6.2 ± 1.0	5.9 ± 1.1	5.5 ± 1.5	.09

*BMI*, body mass index; *SD*, standard deviation; *ASA*, American Society of Anesthesiologists; *COPD*, chronic obstructive pulmonary disease; *PROMIS*, patient reported outcome measurement information system; *ROM*, range of motion; *ANOVA*, analysis of variance.

Bold values indicate *P* <.05.

∗ *P* values for one-way ANOVA (continuous) or chi-squared test or Fisher exact test (categorical).

∗∗ Internal rotation scores were assigned as follows: no motion (0), motion to hip (1), motion to buttock/PSIS/SI, Joint (2), motion to the sacrum (3), motion to L4 to L5 (4), motion to L1 to L3 (5), motion to T8 to T12 (6), and motion to T7 or above (7).

Regarding mean operative time, the obese cohort had a greater surgical duration (100 +/− 40 minutes [obese] vs. 75 +/− 26 minutes [overweight] vs. 76 +/− 29 minutes [normal BMI]; *P* = .001) and an increased incidence of partial rotator cuff tears (RCTs) (60% [obese] vs. 33% [overweight] vs. 27% [normal BMI]; *P* = .009). There were no differences in postoperative PROMIS, CGI, visual analog scale, or ROM between cohorts (*P* > .05). Comprehensive intraoperative findings are available in Table II.

**Table II:** Intraoperative findings and outcomes.

	Overall (n = 142)	BMI < 25 (n = 55)	BMI 25-30 (n = 57)	BMI ≥ 30 (n = 30)	*P* value∗
Procedure duration (min), mean (SD)	80.5 ± 31.7	75.7 ± 28.5	75.3 ± 25.8	99.8 ± 40.0	**.001**
GBL, n (%)	28 (20%)	14 (26%)	10 (18%)	4 (13%)	.4
Average GBL (cm), mean (SD)	0.29 ± 1.72	0.06 ± 0.12	0.64 ± 2.68	0.03 ± 0.08	.1
Hill Sachs lesion, n (%)	79 (56%)	35 (64%)	30 (53%)	14 (47%)	.3
Rotator cuff tear, n (%)	52 (37%)	15 (27%)	19 (33%)	18 (60%)	**.009**
Postoperative PROMIS score					
Pain interference	46 ± 8	45 ± 8	45 ± 8	47 ± 7	.6
Pain intensity	36 ± 7	35 ± 7	36 ± 7	36 ± 6	.7
Upper extremity function	51 ± 10	52 ± 10	51 ± 10	49 ± 10	.4
Postoperative ROM, mean (SD)					
Flexion	165 ± 21	166 ± 21	164 ± 22	143 ± 21	.7
External rotation	59 ± 19	59 ± 18	60 ± 20	56 ± 22	.6
Internal rotation score∗∗∗	6.2 ± 1.1	6.1 ± 1.1	6.3 ± 1.1	6.0 ± 1.4	.3
Postoperative VAS score, mean (SD)	2.5 ± 3.1	2.5 ± 2.8	2.5 ± 3.4	2.6 ± 2.9	.9
Change in PROMIS score∗∗					
Pain interference	−11 ± 9	−11 ± 10	−11 ± 8	−10 ± 9	.8
Pain intensity	−11 ± 9	−11 ± 8	−11 ± 9	−14 ± 8	.1
Upper extremity function	11 ± 12	13 ± 11	12 ± 14	10 ± 11	.1
CGI scale for pain, mean (SD)	1.5 ± 1.0	1.6 ± 1.0	1.4 ± 0.9	1.5 ± 1.1	.5
CGI scale for satisfaction, mean (SD)	1.5 ± 0.9	1.4 ± 0.8	1.5 ± 0.9	1.6 ± 1.0	.8
CGI scale for function, mean (SD)	1.5 ± 1.2	1.6 ± 1.3	1.4 ± 0.8	1.7 ± 1.4	.5

*BMI*, body mass index; *SD*, standard deviation; *GBL*, glenoid bone loss; *PROMIS*, patient reported outcome measurement information system; *ROM*, range of motion; *VAS*, visual analog scale; *CGI*, clinical global impression score; *ANOVA*, analysis of variance.

Bold values indicate *P* <.05.

∗ *P* values for one-way ANOVA (continuous) or chi-squared test or Fisher exact test (categorical).

∗∗ Calculated as difference post- to preoperatively.

∗∗∗ Internal rotation scores were assigned as follows: no motion (0), motion to hip (1), motion to buttock/PSIS/SI, Joint (2), motion to the sacrum (3), motion to L4 to L5 (4), motion to L1 to L3 (5), motion to T8 to T12 (6), and motion to T7 or above (7).

There were no differences in recurrence events and revision surgery rates between the 3 cohorts (*P* > .05). Complications and revision rates are shown in Table III. No significant difference was noted with regards to the number of total complications (3.3% [obese] vs. 5.2% [overweight] vs. 1.8% [normal BMI]; *P* = .378]. There were a total of 5 complications: 3 recurrence of instability (dislocation), 1 postoperative hematoma, and 1 axillary nerve palsy. One patient in each of the normal and overweight BMI cohorts reported an isolated episode of instability (dislocation) with spontaneous reduction treated nonsurgically. One patient in the overweight cohort experienced recurrent dislocations necessitating postoperative revision by a Latarjet procedure 6 months postoperatively. There was one postoperative hematoma reported in the obese cohort treated nonoperatively. Lastly, there was one axillary nerve paresis in the overweight cohort which resolved by 10 months after surgery.

**Table III:** Postoperative complications and revision.

	Overall (n = 142)	BMI < 25 (n = 55)	BMI 25-30 (n = 57)	BMI ≥ 30 (n = 30)	*P* value∗
Any complication, n (%)	5 (3.5)	1 (1.8)	3 (5.2)	1 (3.3)	.3
Dislocation (%)	3 (2.1)	1 (1.8)	2 (3.5)	0 (0)	.5
Nerve palsy (%)	1 (0.7)	0 (0)	1 (1.8)	0 (0)	.2
Hematoma (%)	1 (0.7)	0 (0)	0 (0)	1 (3.3)	.1
Revision surgery, n (%)	1 (1.8)	0 (0)	1 (1.8)	0 (0)	.1

*BMI*, body mass index; *SD*, standard deviation; *ANOVA*, analysis of variance.

∗ *P* values for one-way ANOVA (continuous) or chi-squared test or Fisher exact test (categorical).

After controlling for confounding factors including age, sex, smoking status and comorbidities, multivariable linear regression analysis demonstrated obesity to be associated with poorer improvement in pre- to postoperative P-UE scores (obese vs. normal: Β −4.97 [−9.42, −0.53]; *P* = .029) and a longer procedure duration (obese vs. normal: Β 20.38 [6.41, 34.36]; *P* = .005). A full complete list of outcomes and their associated odds ratio or Beta coefficient are shown in Table IV.

**Table IV:** Multivariable regression results for body mass index group as predictor of postoperative outcomes.∗

Outcome	Odds ratio or β coefficient with 95% CI	*P* value
Postoperative PROMIS score		
Pain interference	Obese vs. normal: 2.62 [−1.26, 6.27]	.1
	Overweight vs. normal: 0.65 [−2.19, 3.51]	.6
Pain intensity	Obese vs. normal: 0.79 [−2.39, 3.98]	.6
	Overweight vs. normal: 0.38 [−2.10, 2.88]	.7
Upper extremity function	Obese vs. normal: −1.43 [−5.03, 2.16]	.4
	Overweight vs. normal: −1.35 [−5.95, 3.24]	.5
Change in PROMIS score∗		
Pain interference	Obese vs. normal: 2.52 [−1.96, 7.01]	.2
	Overweight vs. normal: 1.15 [−2.36, 4.65]	.5
Pain intensity	Obese vs. normal: −0.61 [−4.71, 3.49]	.7
	Overweight vs. normal: 0.91 [−2.29, 4.13]	.5
Upper extremity function	Obese vs. normal: −4.97 [−9.42, −0.53]	**.029**
	Overweight vs. normal: −0.95 [−6.64, 4.73]	.7
CGI scale scores		
CGI scale for pain	Obese vs. normal: −0.053 [−0.53, 0.43]	.8
	Overweight vs. normal: −0.19 [−0.57, 0.19]	.3
CGI scale for function	Obese vs. normal: 0.10 [−0.48, 0.68]	.7
	Overweight vs. normal: −0.17 [−0.63, 0.27]	.4
Satisfaction	Obese vs. normal: 0.18 [−0.25, 0.62]	.8
	Overweight vs. normal: 0.07 [−0.27, 0.41]	.6
Procedure duration		
	Obese vs. normal: 20.38 [6.41, 34.36]	**.005**
	Overweight vs. normal: −0.97 [−11.90, 9.96]	.8
Concomitant lesion		
Hill Sachs lesion	Obese vs. normal: 0.79 [0.36, 1.76]	.8
	Overweight vs. normal: 0.71 [0.26, 1.94]	.7
Rotator cuff tear	Obese vs. normal: 3.22 [1.13, 9.21]	**.028**
	Overweight vs. normal: 1.34 [0.57, 3.12]	.4
Postoperative complication		
	Obese vs. normal: 4.04 [0.41, 39.99]	.2
	Overweight vs. normal: 2.48 [0.12, 52.33]	.5

*CI*, confidence interval; *PROMIS*, patient reported outcomes measurement information system; *CGI*, clinical global impression score.

Bold values indicate *P* <.05.

Logistic and linear regression analyses used for categorical and continuous outcomes, respectively.

∗ Calculated as difference post- to preoperatively.

## Discussion

The purpose of this study was to examine the impact of obesity on the 1-year minimal outcomes and rate of postoperative complications in patients undergoing ABR. We found that obesity (BMI > 30) was associated with longer mean procedure times. Despite these differences, obesity did not increase the rate of recurrence and revision surgery by 1 year following ABR (*P* > .05).

Reflective of its overall prevalence and anticipated increase in incidence over the next several decades, obesity is one of the most common and important health concerns in the United States.^9^, ^10^, ^11^, ^12^, ^13^, ^14^, ^15^ As a result, shoulder surgeons are expected to treat the full spectrum of shoulder pathology, while maintaining a high standard of care in an ever-progressing obese population. As with all patients, obese patients are at risk for developing anterior shoulder instability. Whether it be by ligamentous laxity or traumatic injury, this is the first study to evaluate by what extent obesity impacts surgical outcomes and specific complications after ABR. Identification of these risks is paramount and can better inform surgeons as to operative success rates, better gauge patient expectations, and potentially impact the operative vs. nonoperative management.

Interestingly, our study found that obesity did not increase the risk of instability recurrence or result in poor patient reported outcome measures (PROMs) after ABR. It is important to note that the overall 1-year complication rates, specifically for dislocation and revision, were low in this study. This may indicate that there may have been insufficient power to detect a difference between groups, particularly if the effect size of obesity on complication rate is low. Our study findings parallel that of literature investigating rotator cuff repair (RCR), which has shown that obese patients experience noninferior outcomes compared to nonobese patients after arthroscopic RCR, total shoulder arthroplasty (TSA), and other orthopedic procedures.^11^^,^^14^^,^^16^^,^^26^^,^^31^^,^^43^

Though the literature regarding BMI and its impact on the outcomes following ABR is limited, BMI has been assessed for its influence on patient outcomes following other shoulder procedures (open and arthroscopic procedures).^16^^,^^31^^,^^43^ Studies have demonstrated increased BMI does not increase the risk of complications following TSA, result in worse PROMS, or decreased postoperative ROM.^31^^,^^43^ Similarly, patients with elevated BMIs were found to not be associated with worse pre- to postoperative differences in PROM scores following RCR^16^ Although not equivalent to ABR, TSA and RCR for patients with higher BMI have demonstrated good postoperative PROMs and no increase in complications.^16^^,^^31^^,^^43^

While diabetes mellitus and age have been reported as an associated risk factor for RCTs, there is controversy regarding a direct association between elevated BMI and these tears.^8^^,^^29^^,^^38^^,^^42^^,^^47^ Although age-related RCTs are most prevalent in patients older than 60 years, recurrent anterior dislocation in patients over the age of 40 years is associated with concomitant RCTs.^24^ Our results have demonstrated elevated BMI (BMI > 30 (60%) vs. BMI < 25 (27%); *P* = .009) to be associated with an increased propensity for concurrent partial RCTs. Although the obese cohort reflects a higher age (BMI > 30: 42 +/− 13 years old vs. BMI < 25: 31 +/− 12 years old), we can appreciate a large standard deviation among ages resulting in a high level of overlap across the cohorts mitigating the risk of age on RCT which has demonstrated a higher propensity in patients with advancing age.^47^ Similar conclusions have been drawn by Macchi et al, with data demonstrating obese patients have a greater risk of rotator cuff tendinopathy (odds ratio [OR]: 1.25, 95% confidence interval [CI]: 1.12-1.40) and RCTs (OR: 2.35, 95% CI: 1.62-3.40; *P* < .001).^25^ Although none of the partial RCTs within our study required immediate repair, clinicians should be aware of this relationship in the assessment of obese patients with dislocation events and potential for worsening of pain upon presentation.^16^ Additionally, there should be a high index of suspicion for RTC tear in obese patients, even at ages younger than one would expect a RTC tear after dislocation.^17^

In our study, we found obesity to be associated with an increased mean operative time, a finding consistent with that of other orthopedic procedures including reverse TSA, peritrochanteric femur fractures, anterior cruciate ligament reconstructions, and distal radius fractures.^12^^,^^18^^,^^28^^,^^32^^,^^37^^,^^44^ Compared to those with a normal BMI, obese patients required 132% more time to complete surgery. This may be indicative of difficulty with positioning obese patients, creating portals, as well as the potential need for special instruments.^27^ Additionally, shoulder swelling during surgery is more difficult to manage in obese patients with decreased excursion of the cannula and extrusion of cannula within a portal. These factors may explain the increased mean operative times for obese patients. It is important for surgeons to keep in mind the potential additional time necessary when booking such cases and determining workflow. While longer operative times have been associated with higher risks of postoperative complications, especially infection, our study did not demonstrate a higher risk of postoperative complications in the obese cohort.^10^

From an administrative perspective, the growing prevalence of obesity and longer operative times in these populations has potential to impact health-care expenses. As capitated insurance models such as bundled payments and fee-for-service continue to grow in popularity, clinicians are required to play an increasingly active role in cost-mitigation. Operating room costs account for a significant portion of surgical care expenses and it is important for surgeons to be cognizant of factors increasing operating time, and thus operating costs.^34^ Previous studies have shown that the cost per minute of operating room time averaged $62, ranging from $22 to $133.^35^ Utilizing an average operating room cost per minute of $62, our study estimates that obese patients incur an average additional operating room cost of $1494 as compared to normal weight patients. Additionally, this can affect the flow and number of cases during a given operating day. Notably, these increased costs are not covered by increased reimbursement by third parties, thus contributing to an increasing financial burden on the surgical facility.^35^ Further research is needed to determine the economic burden of obesity in the surgical setting.

There are several limitations to this study. Although located in an extremely diverse metropolitan location, our study was performed at a single institution and limited to English-only speaking patients due to the PROM surveys that were administered. These factors may limit study generalizability. This study utilized our institution's database and improper charting and selection bias may have influenced the results. We attempted to mitigate the effect of selection bias on the results by controlling for all significant differences in baseline characteristics. Although all patients demonstrated instability necessitating operative intervention, we were unable to determine if any impact existed regarding the time from first dislocation till the time of surgery or the impact the number of dislocations prior to surgery may have had on if a patient underwent surgery. We believe this would be an opportunity for future research. Lastly, the overall incidence of postoperative complications was relatively small and the effect size of obesity on complication rate may be relatively small, increasing the chance of a type II error.

## Conclusion

Obesity is an independent risk factor for longer operative times but does not confer a higher risk of recurrent instability, revision surgery, or lower outcome scores 1 year following ABR.

## Disclaimers

Funding: No funding was disclosed by the authors.

Conflicts of interest: The authors, their immediate families, and any research foundations with which they are affiliated have not received any financial payments or other benefits from any commercial entity related to the subject of this article.
